# Supplementary material for: Identifying Extrinsic versus Intrinsic Drivers of Variation in Cell Behavior in Human iPSC Lines from Healthy Donors
Source: Cell Rep. 2019 Feb 19;26(8):2078–2087.e3. doi: 10.1016/j.celrep.2019.01.094 (PMC6381787; doi:10.1016/j.celrep.2019.01.094)
Supplement: Document S1. Figures S1–S4 and Table S2 [file mmc1.pdf]

Cell Reports, Volume *26*

Supplemental Information

Identifying Extrinsic versus Intrinsic Drivers of Variation in Cell Behavior in Human iPSC Lines from Healthy Donors

Alessandra Vigilante, Anna Laddach, Nathalie Moens, Ruta Meleckyte, Andreas Leha, Arsham Ghahramani, Oliver J. Culley, Annie Kathuria, Chloe Hurling, Alice Vickers, Erika Wiseman, Mukul Tewary, Peter W. Zandstra, HipSci Consortium, Richard Durbin, Franca Fraternali, Oliver Stegle, Ewan Birney, Nicholas M. Luscombe, Davide Danovi, and Fiona M. Watt

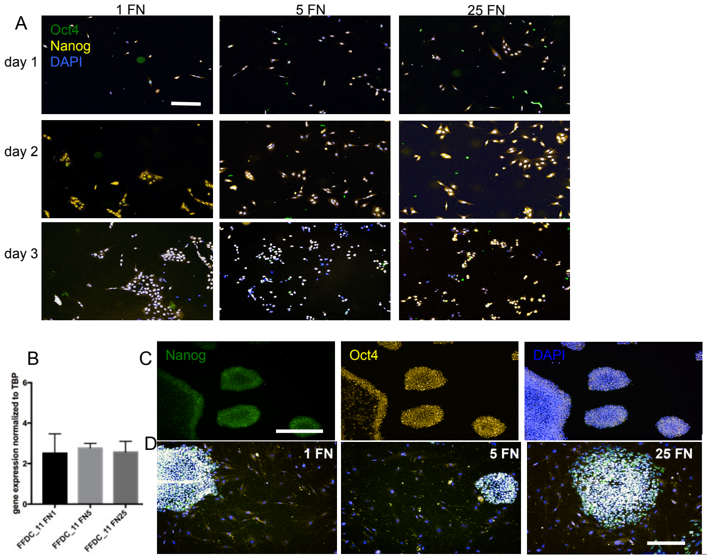

Figure S1. Related to Figure 1. A. Antibody labelling of A1ATD-iPSC patient 1 cells for OCT4 and NANOG, with DAPI counterstain, after plating on the FN concentrations shown. Cells were fixed after 1, 2 or 3 days. Scale bar: 150 µm. **B.** Q-PCR of OCT4 expression in FFDC_11 cells 24h after plating on the FN concentrations shown. Means ± SD of triplicate wells. **C, D.** A1ATD-iPSC patient 1 cells (C) or CTR M205 cells (D) were plated on vitronectin (C) or the FN concentrations shown (D) for 24h and then harvested and replated on feeders (D) or under feeder-free conditions (C). Two days later the cultures were fixed and labelled with antibodies to NANOG and OCT4 with DAPI counterstain. Representative colonies are shown. The same colonies are shown in each panel of (C). Scale bar: 150 µm.

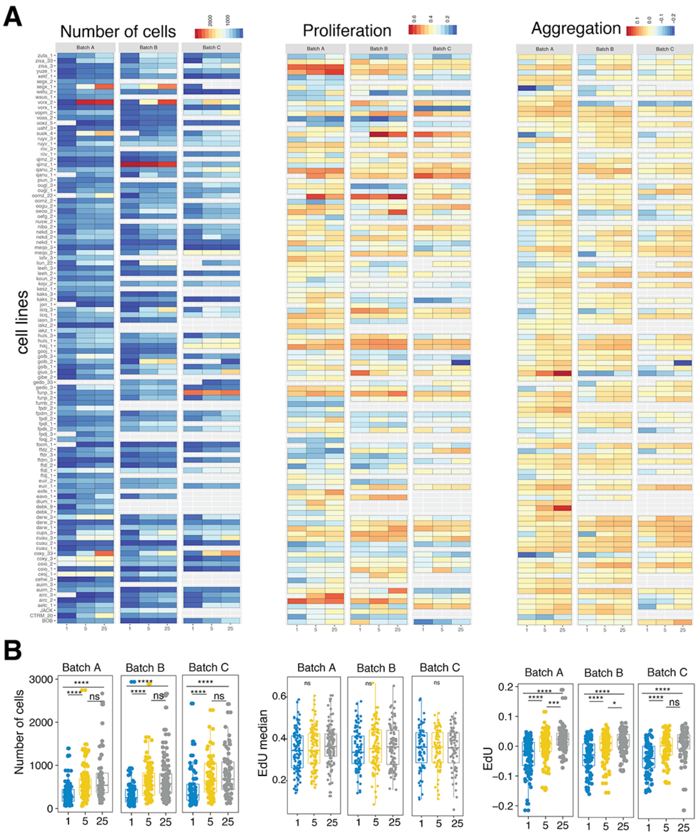

Figure S2. Related to Figure 1. A. Heatmaps of mean values for three phenotypic measurements: number of cells, proliferation (EdU incorporation) and cell aggregation (clump size) for each cell line on three fibronectin concentrations in three independent experiments (batches). Grey boxes correspond to experiments not performed. **B.** Boxplots of mean values for the same three phenotypic measurements on three fibronectin concentrations in three biological replicates (batches). Each dot is one cell line. Asterisks (**** P ≤ 0.0001; *** P≤ 0.001; ** P ≤ 0.01; * P ≤ 0.05; ns not significant) represent significance values from pairwise t-tests performed between each condition.

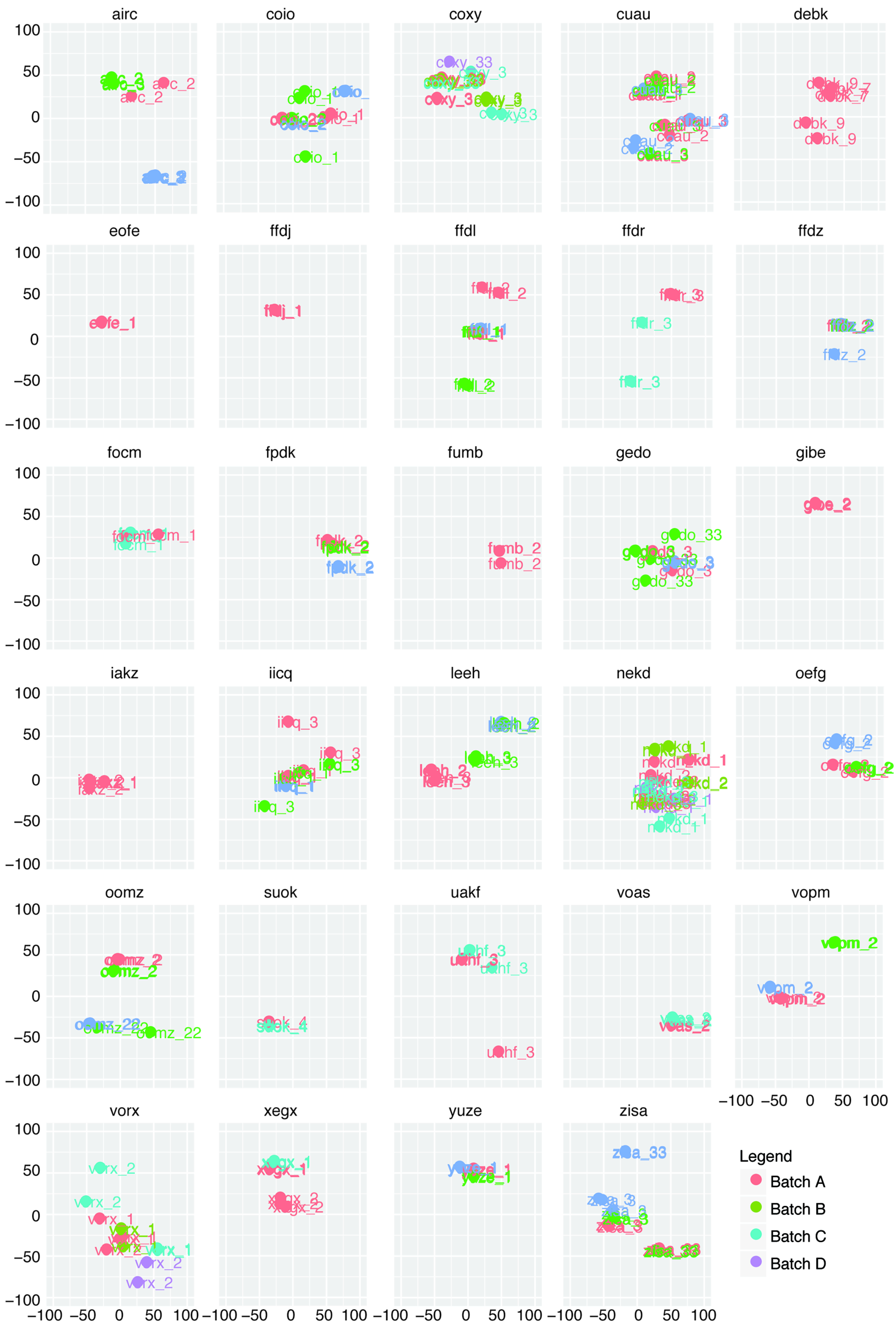

Figure S3. Related to Figure 1. t-SNE plots for each outlier cell line representing the reproducibility (cell area phenotype) of 3 technical replicates per batch, and variation between batches. Each dot within each plot is a technical replicate. Colors represent individual batches. Different lines from the same donor are also indicated.

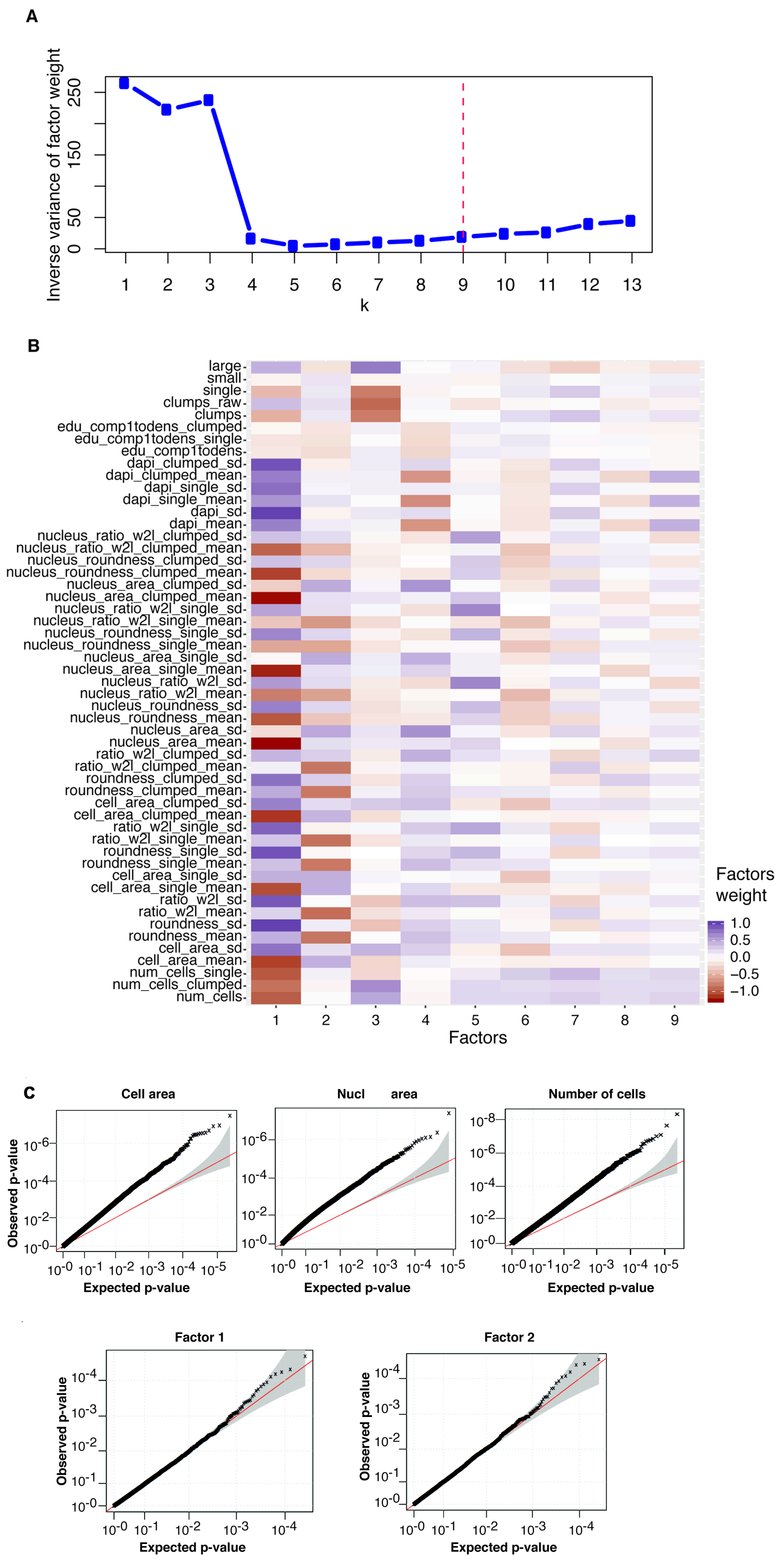

Figure S4. Related to Figure 3. A. Plot of inverse variance of PEER factor weights (y axis) against k number (x axis) (usually observed as an "elbow"). **B.** Heatmap showing weight of each raw phenotype onto each of the 9 PEER factors. **C.** Q-Q plots in which the -log10 of p-values observed for the correlation analyses using raw data (upper panels) or the PEER factors (lower panels) are plotted against the theoretical -log10 p-values expected under the null hypothesis (red line). Each dot represents one gene.

Table S2

Cell area	Clumped cells	mean	1
		standard deviation	2
	Single cells	mean	3
		standard deviation	4
	All cells	mean	5
		standard deviation	6
Cell ratio w/l	Clumped cells	mean	7
		standard deviation	8
	Single cells	mean	9
		standard deviation	10
	All cells	mean	11
		standard deviation	12
Cell roundness	Clumped cells	mean	13
		standard deviation	14
	Single cells	mean	15
		standard deviation	16
	All cells	mean	17
		standard deviation	18
Nucleus area	Clumped cells	mean	19
		standard deviation	20
	Single cells	mean	21
		standard deviation	22
	All cells	mean	23
		standard deviation	24
Nucleus ratio w/l	Clumped cells	mean	25
		standard deviation	26
	Single cells	mean	27
		standard deviation	28
	All cells	mean	29
		standard deviation	30
Nucleus roundness	Clumped cells	mean	31
		standard deviation	32
	Single cells	mean	33
		standard deviation	34
	All cells	mean	35
		standard deviation	36
DAPI	Clumped cells	mean	37
		standard deviation	38
	Single cells	mean	39
		standard deviation	40
	All cells	mean	41
		standard deviation	42
EdU	Clumped cells	mean	43
		standard deviation	44
	Single cells	mean	45
		standard deviation	46
	All cells	mean	47
		standard deviation	48
Number of cells	Clumped cells		49
	Single cells		50
	All cells		51
Clumps			52

Table S2. Related to Figure 1. List of all 52 cell features analysed.
