# Supplementary material for: Identifying Extrinsic versus Intrinsic Drivers of Variation in Cell Behavior in Human iPSC Lines from Healthy Donors
Source: Cell Rep. 2019 Feb 19;26(8):2078–2087.e3. doi: 10.1016/j.celrep.2019.01.094 (PMC6381787; doi:10.1016/j.celrep.2019.01.094)
Supplement: Document S2. Article plus Supplemental Information [file mmc7.pdf]

Article

Cell Reports

# Identifying Extrinsic versus Intrinsic Drivers of Variation in Cell Behavior in Human iPSC Lines from Healthy Donors

## Graphical Abstract

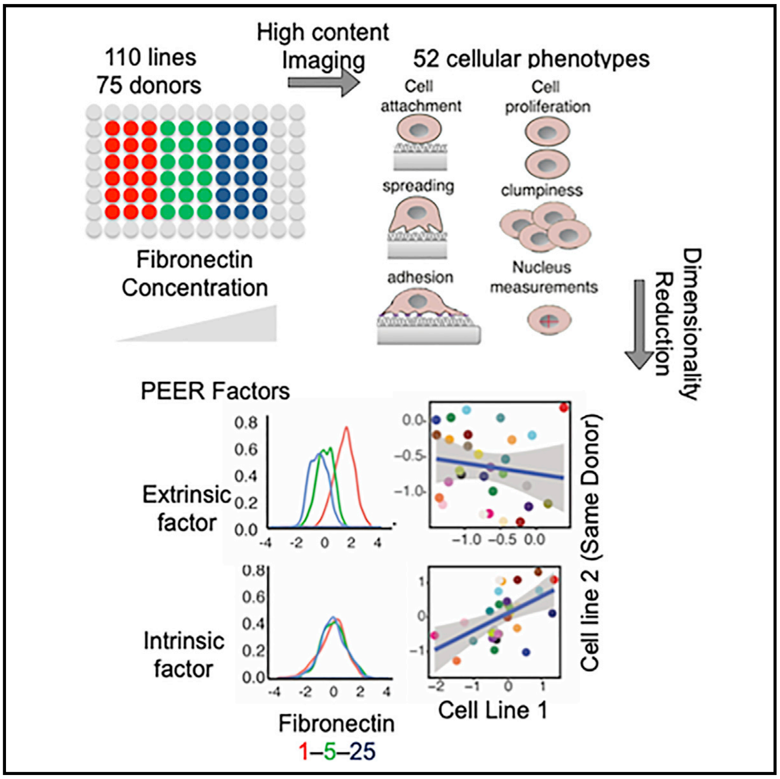



## Authors

Alessandra Vigilante, Anna Laddach, Nathalie Moens, ..., Nicholas M. Luscombe, Davide Danovi, Fiona M. Watt

## Correspondence

davide.danovi@kcl.ac.uk (D.D.),
fiona.watt@kcl.ac.uk (F.M.W.)



## In Brief

Cell behavior reflects both the intrinsic state of the cell and extrinsic signals it receives from its microenvironment. By integrating genomic, gene expression, and cell biology datasets from a large number of human iPSCs from healthy donors, Vigilante et al. show how genetic variation contributes to phenotypic variation.


## Highlights

- We used human iPSCs to investigate how genetic variations affect cell behavior
- We integrate genomic, gene expression, and cell biology datasets
- We identify many mRNAs that correlated with modal cell behavior
- Outlier cell behavior correlates with genes containing rare deleterious SNVs

Vigilante et al., 2019, Cell Reports *26*, 2078–2087
February 19, 2019 © 2019 The Authors.
https://doi.org/10.1016/j.celrep.2019.01.094

OPEN ACCESS CellPress

Cell Reports
Article

# Identifying Extrinsic versus Intrinsic Drivers of Variation in Cell Behavior in Human iPSC Lines from Healthy Donors

Alessandra Vigilante,[1,2,4,8] Anna Laddach,[5] Nathalie Moens,[1,10] Ruta Meleckyte,[1,11] Andreas Leha,[3,12] Arsham Ghahramani,[1,4] Oliver J. Culley,[1] Annie Kathuria,[1] Chloe Hurling,[1] Alice Vickers,[1] Erika Wiseman,[1] Mukul Tewary,[1,6,7] Peter W. Zandstra,[6,7] HipSci Consortium, Richard Durbin,[3,9] Franca Fraternali,[5] Oliver Stegle,[2] Ewan Birney,[2] Nicholas M. Luscombe,[4,8] Davide Danovi,[1,*] and Fiona M. Watt[1,13,*]

[1]Centre for Stem Cells and Regenerative Medicine, King's College London, Floor 28, Tower Wing, Guy's Hospital, Great Maze Pond, London SE1 9RT, UK
[2]European Molecular Biology Laboratory, European Bioinformatics Institute, Wellcome Genome Campus, Hinxton, Cambridge CB10 1SA, UK
[3]Wellcome Sanger Institute, Wellcome Genome Campus, Hinxton CB10 1SA, UK
[4]The Francis Crick Institute, 1 Midland Road, London NW1 1AT, UK
[5]Randall Division, King's College London, New Hunts House, Great Maze Pond, London SE1 9RT, UK
[6]School of Biomedical Engineering, The University of British Columbia, 2222 Health Sciences Mall, Vancouver, BC V6T 1Z3, Canada
[7]Michael Smith Laboratories, The University of British Columbia, 2185 East Mall, Vancouver, BC V6T 1Z4, Canada
[8]UCL Genetics Institute, Department of Genetics, Evolution and Environment, University College London, Gower Street, London WC1E 6BT, UK
[9]Department of Genetics, University of Cambridge, Downing Street, Cambridge CB2 3EH, UK
[10]Present address: Glaxo Smith Kline, Gunnels Wood Road, Stevenage, Herts SG1 2NY, UK
[11]Present address: Sobell Department, University College London Institute of Neurology, Queen Square House, Queen Square, London WC1N 3GB, UK
[12]Present address: Department of Medical Statistics, University Medical Center Göttingen, Georg-August-Universität, Humboldtallee 32, 37073 Göttingen, Germany
[13]Lead Contact
*Correspondence: davide.danovi@kcl.ac.uk (D.D.), fiona.watt@kcl.ac.uk (F.M.W.)
https://doi.org/10.1016/j.celrep.2019.01.094

## SUMMARY

**Large cohorts of human induced pluripotent stem cells (iPSCs) from healthy donors are a potentially powerful tool for investigating the relationship between genetic variants and cellular behavior. Here, we integrate high content imaging of cell shape, proliferation, and other phenotypes with gene expression and DNA sequence datasets from over 100 human iPSC lines. By applying a dimensionality reduction approach, Probabilistic Estimation of Expression Residuals (PEER), we extracted factors that captured the effects of intrinsic (genetic concordance between different cell lines from the same donor) and extrinsic (cell responses to different fibronectin concentrations) conditions. We identify genes that correlate in expression with intrinsic and extrinsic PEER factors and associate outlier cell behavior with genes containing rare deleterious non-synonymous SNVs. Our study, thus, establishes a strategy for examining the genetic basis of inter-individual variability in cell behavior.**

## INTRODUCTION

Now that the applications of human induced pluripotent stem cells (hiPSCs) for disease modeling and drug discovery are well established, attention is turning to the creation of large cohorts of hiPSCs from healthy donors. These offer a unique opportunity to examine common genetic variants and their effects on gene expression and cellular phenotypes (Warren et al., 2017; Pashos et al., 2017; Carcamo-Orive et al., 2017; DeBoever et al., 2017; Kilpinen et al., 2017). Genome-wide association studies (GWASs) and quantitative trait locus (QTL) studies can be used to correlate SNPs and other genetic variants with quantitative phenotypes (Panopoulos et al., 2017). As a contribution to this effort, we recently described the generation and characterization of over 700 open access hiPSC lines derived from 301 healthy donors through the Human Induced Pluripotent Stem Cell Initiative (HipSci) (Kilpinen et al., 2017; www.hipsci.org). In addition to creating a comprehensive reference map of common regulatory variants affecting the transcriptome of hiPSCs, we performed quantitative assays of cell morphology and demonstrated a donor contribution in the range of 8%–23% to the observed variation (Kilpinen et al., 2017). In the present study, we set out to identify genetic drivers of cell behavior.

Previous attempts using lymphoblastoid cell lines to link genetics to *in vitro* phenotypes have had limited success (Choy et al., 2008; Jack et al., 2014). In that context, confounding effects included Epstein Barr virus (EBV) viral transformation, the small number of lines analyzed, variable cell culture conditions, and line-to-line variation in proliferation rate. These factors decrease the power to detect true relationships between DNA variation and cellular traits (Choy et al., 2008). In contrast, we have access to a large number of hiPSC lines derived using

 © 2019 The Authors.
This is an open access article under the CC BY license (http://creativecommons.org/licenses/by/4.0/).

standard protocols from healthy volunteers, including multiple lines from the same donor. In addition, HipSci lines present a substantially lower number of genetic aberrations than reported for previous collections (Kilpinen et al., 2017; Laurent et al., 2011). Cells are examined over a limited number of passages, and cell properties are evaluated at single-cell resolution during a short time frame, using high-throughput quantitative readouts of cell behavior.

Stem cell behavior reflects both the intrinsic state of the cell (Choi et al., 2015; Kyttälä et al., 2016) and the extrinsic signals it receives from its local microenvironment, or niche (Lane et al., 2014; Reimer et al., 2016). We hypothesized that subjecting cells to different environmental stimuli increases the likelihood of uncovering links between genotype and cell behavior. For that reason, we seeded cells on different concentrations of the extracellular matrix (ECM) protein fibronectin that support cell spreading to differing extents and assayed the behavior of single cells and cells in contact with their neighbors. We took a ''cell observatory'' approach, using high-throughput, high-content imaging to gather data from millions of cells 24 h after seeding. We then applied a multidimensional reduction method, Probabilistic Estimation of Expression Residuals (PEER) (Stegle et al., 2012), to reveal the underlying structure in the dataset and correlated cell behavior with the expression of a subset of genes and the presence of rare deleterious non-synonymous single nucleotide variants (nsSNVs). The strategy we have developed bridges the gap between genetic and transcript variation on the one hand and cell phenotype on the other, and should be of widespread utility in exploring the genetic basis of inter-individual variability in cell behavior.

## RESULTS

### Generation and Characterization of the Lines

We analyzed 110 cell lines, 107 from the HipSci resource (Kilpinen et al., 2017) and 3 non-HipSci control lines (Table S1). Of these, 99 lines were reprogrammed by Sendai virus and 11 using episomal vectors. A total of 100 lines came from 65 healthy research volunteers; thus, several lines were derived from different clones from the same donor. Seven lines came from 7 individuals with Bardet-Biedl syndrome. Out of the total, 102 of the lines were derived from skin fibroblasts, 6 from peripheral blood monocytes and 2 from hair follicles. Lines were subjected to the quality controls specified within the HipSci production pipeline, including high PluriTest (Stem Cell Assays) scores and the ability to differentiate along the three embryonic germ layers. All the cell lines were reprogrammed on feeders, and all but 6 lines were cultured on feeders prior to phenotypic analysis (Table S1). Most cells were examined between passages 15 and 45 (Table S1).

### Cell Behavior Assays

To quantitate cell behavior at single-cell resolution, we used the high-content imaging platform that we described previously (Leha et al., 2016). Cells were disaggregated and resuspended in the presence of 10-μM Rho-associated protein kinase (ROCK) inhibitor to minimize cell clumping. In order to vary the extrinsic conditions for cell adhesion and spreading, cells were seeded on 96-well plates coated with 3 different concentrations of fibronectin, namely, 1, 5, and 25 μg/mL (Fn1, Fn5, and Fn25, respectively), with Fn1 representing a suboptimal concentration for cell attachment and spreading. After 24 h of culture in the presence of the ROCK inhibitor, cells were labeled with 5-ethynyl-2′-deoxyuridine (EdU) for 30 min (to detect proliferative cells), fixed, and stained with 4′,6-diamidino-2-phenylindole (DAPI) (to visualize nuclei) and CellMask (to visualize cytoplasm) (Figure 1A; Figure S1A). qPCR (Figure S1B) and antibody labeling (Figure S1A) confirmed that pluripotency was maintained at 24 h, regardless of FN concentration. In addition, when cells were harvested from FN and replated, they were able to form colonies containing a majority of Oct4- and NANOG-positive cells (Figures S1C and S1D).

Three replicate wells were seeded per cell line, and each cell line was analyzed in up to three independent experiments. Wells containing technical triplicates of each fibronectin concentration were randomized per column (e.g., 1-5-25; 5-25-1; 25-1-5) to obviate edge and position effects. Technical replicates of the same cell line were randomized in rows and one line, previously reported as A1ATD-iPSC patient 1 (Rashid et al., 2010), was included to control for biological variation between experiments.

From each of approximately 2 million cells, we extracted a total of 11 measurements, 10 per cell (i.e., object-based), plus the number of cells per well (i.e., well-based) (Figures 1B-1D). Cell features included the derived area, roundness, and width to length ratio of each cell and each nucleus. We also determined clump size, a context feature representing the number of cells in a group that were in contact with one another. We then measured the features of individual solitary cells and individual cells within a group (Figures 1B–1D). Some features were positively correlated with one another, such as cell area and nuclear area, whereas in other cases, such as cell area and cell roundness, there was an inverse correlation (Figure 1C). The phenotypic features were processed as described previously (Leha et al., 2016), i.e., well-based measurements were normalized in value (log10 or square transformation) and aggregated across the cells in each well by taking the average and standard deviation. For EdU incorporation, median pixel intensity raw values per cell were used to extract a well-based measure of the fraction of EdU-positive cells (Leha et al., 2016). This resulted in a final list of 52 features (Table S2).

The scale and complexity of the cell phenotype dataset is illustrated in Figure 1F, in which the mean value of cell area is represented for all cell lines, for three fibronectin concentrations and three biological replicates (batches; gray bars indicate replicates that were not performed). This highlights the variance we observed between replicate experiments. It also reveals the extent of variability for cell lines derived from the same donor, denoted by a common 4-letter code (Figure 1F). It shows a consistent effect of fibronectin concentration on cell behavior, with cells exhibiting a smaller spread area on the lowest concentration (see also Figure 1D). Figure 1E shows that fibronectin concentration has a significant effect on cell area and that the effect is greater than the variance between cell lines and between biological replicates. Similar results were obtained for other raw phenotypic features (Figure S2). We conclude that FN concentration, which is an extrinsic or environmental factor, influences cell behavior regardless of the donor origin of each cell line.

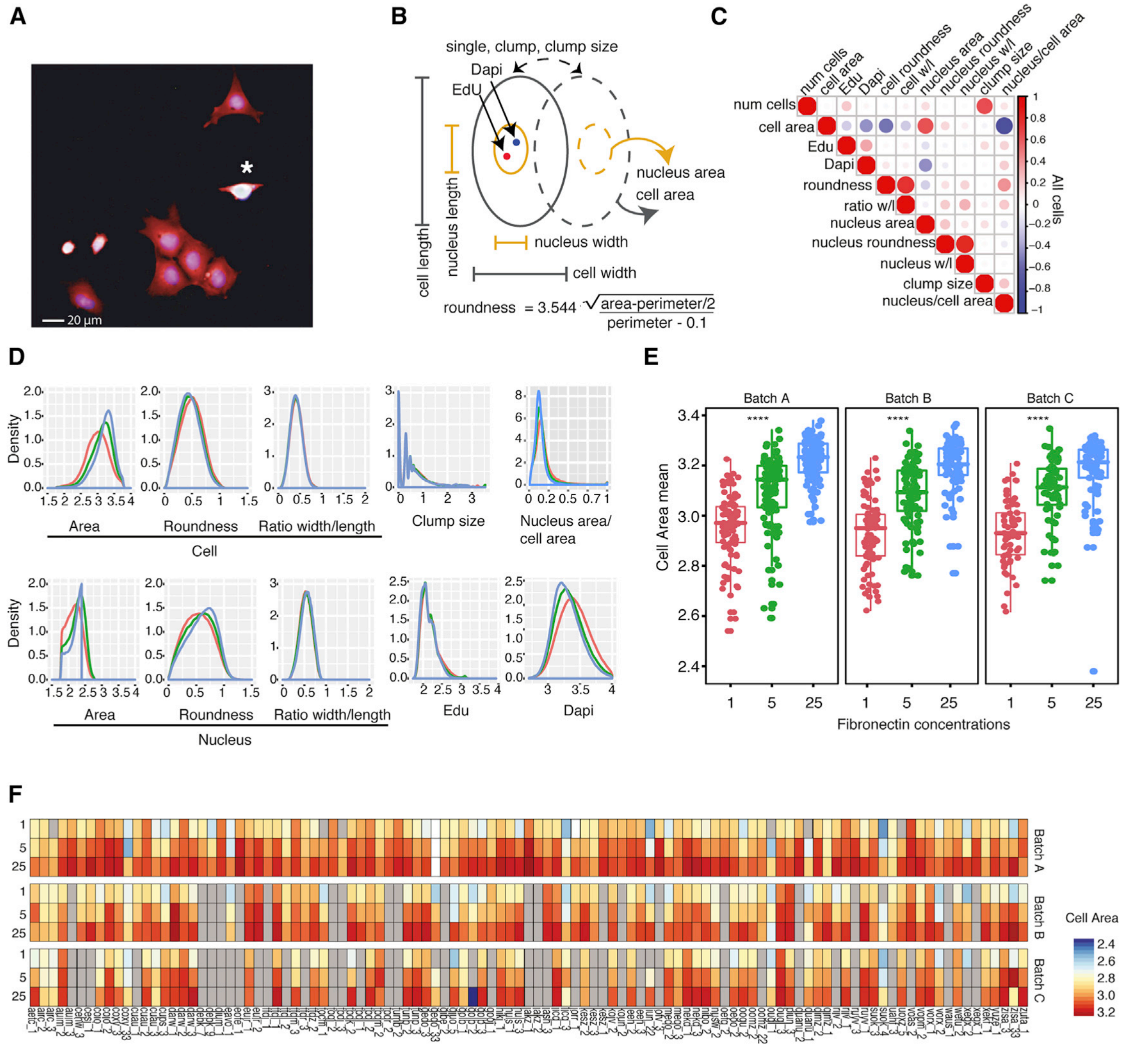

**Figure 1. Description of Phenotypic Dataset**

(A) Microscopic image showing cells 24 h after plating. Red: cell mask (cytoplasm); white: EdU incorporation (DNA synthesis, one EdU+ cell marked with asterisk); blue: DAPI (nuclei). Scale bar: 20 μm.

(B) Schematic of phenotypic features measured in this study.

(C) Correlation of different phenotypic measurements in all cells.

(D) Distribution of main phenotypic features of all cell lines on three fibronectin concentrations (Fn1, red; Fn5, green; Fn25, blue). y axis: density measurements represent the cell number distributions.

(E) Boxplots of mean cell area on three fibronectin concentrations in three biological replicates (batches). Each dot is one cell line. Asterisks (****p $\leq$ 0.0001) represent significance values from pairwise t tests performed between each condition.

(F) Heatmap of mean cell area measurements for each cell line on three fibronectin concentrations in three independent experiments. Grey boxes correspond to replicates not performed.

## Identification of Outlier Cell Lines

Having established that FN concentration, an extrinsic factor, influences cell behavior, we next examined whether individual cell lines exhibited outlier FN responses as a potential route to exploring genetic (intrinsic) contributions to cell phenotype. Outlier cell lines were defined as lines that deviated significantly from modal phenotypic values. To identify them, we performed a Kolmogorov-Smirnov test of the distributions of each raw phenotypic feature for each individual cell line compared to all cell lines (Figure 2). We arbitrarily defined outliers as cell lines with a statistic (D) value above the 95th percentile. Out of the 110 lines analyzed, 36 lines from 30 donors exhibited outlier behavior for one or more phenotypic feature (Figure 2; Figure S3).

In support of a genetic contribution to outlier cell behavior, in several cases two independent lines from the same donor exhibited the same outlier behavior. For example iakz_1 and iakz_2 were outliers for cell roundness, whereas airc_2 and airc_3 were outliers for DAPI nuclear staining intensity. In addition, where two phenotypes were positively or negatively

OPEN
ACCESS
CellPress

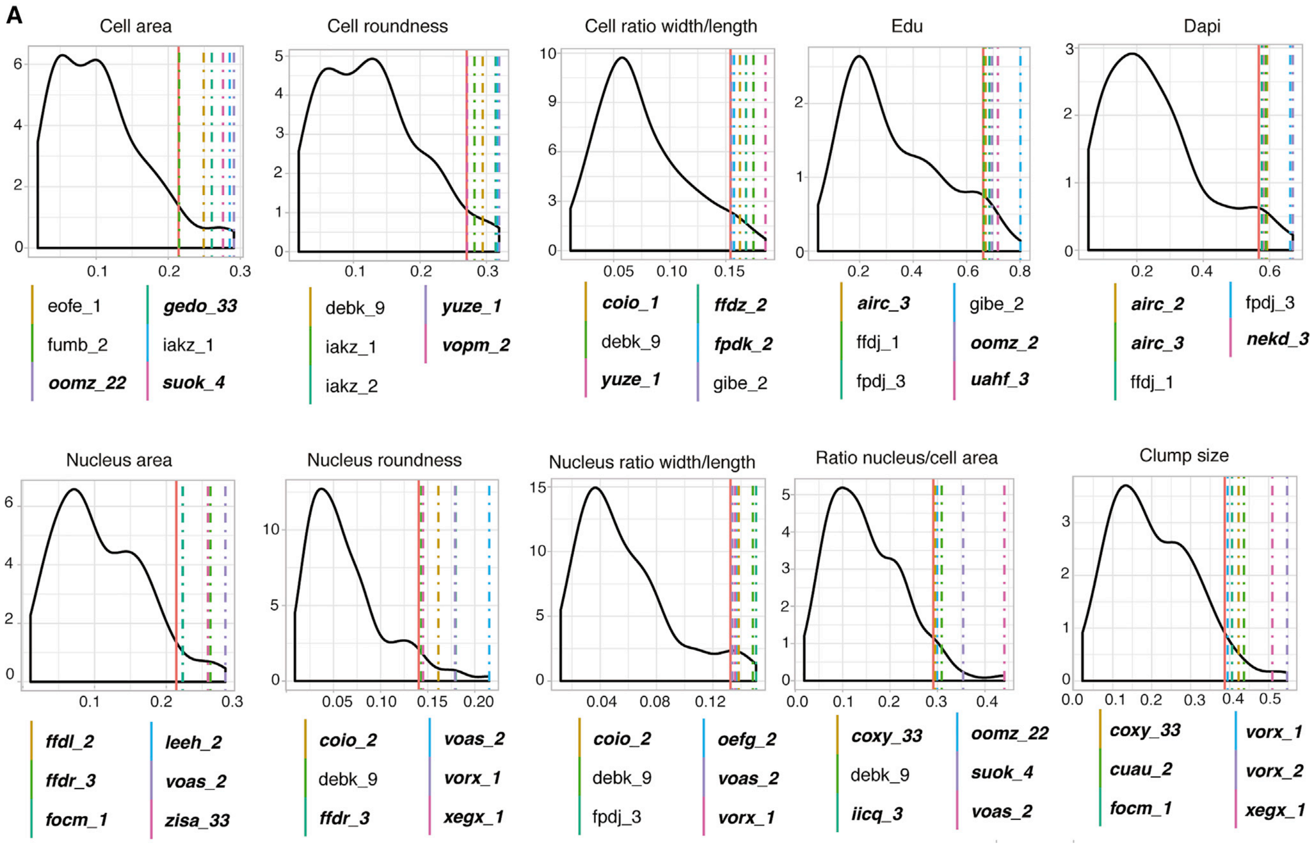


**Figure 2. Identification of Outlier Cell Lines for Individual Phenotypes**

The distribution of the Kolmogorov-Smirnov statistic (D) obtained by performing the Kolmogorov-Smirnov test of the distributions of each raw phenotypic feature for each individual cell line compared to all cell lines. 95$^{th}$ percentile threshold is shown as a red line together with values of individual outlier lines (color coded). Lines listed in italic bold correspond to lines having outlier measurements in more than one batch.

correlated (e.g., cell area and cell roundness), some cell lines were outliers in both categories (e.g., iakz_1).

## Applying PEER to Discover Determinants of Variation in Cell Behavior

In order to explore how extrinsic (i.e., different fibronectin concentrations), intrinsic (i.e., cell line donor specific), and technical or biological components (covariates) contributed to the observed variation in cell phenotypes, we applied a dimensionality reduction approach called Probabilistic Estimation of Expression Residuals (PEER) (Stegle et al., 2012). PEER is a software package that implements Bayesian statistical models that improve the sensitivity and interpretability of genetic associations in population-scale data. It takes as input gene transcript profiles and covariates from a set of individuals and then outputs hidden factors (PEER factors) that explain the expression variability. Many previous studies have demonstrated the importance of accounting for hidden factors to achieve a stronger statistical discrimination signal (Leek and Storey, 2007; Stegle et al., 2008; Kang et al., 2008). Here, we have applied PEER to multidimensional reduction of cell phenotypic data.

In our analysis, we input the 52 phenotypic measurements (Table S2), the 3 covariates (i.e., fibronectin concentrations, experimental replicates, and individual donors), and the estimated total number of unobserved factors (k). To obtain this number, the PEER analysis was repeated several times with a range of values of k (from 1 to 13), and for each k the inverse of the variance of the factor weight was calculated with automatic relevance determination (ARD) (Stegle et al., 2012) (Figure S4A). The plot of the inverse variance of factor weights against the k number (usually observed as an "elbow") shows that above k = 9, the inverse variance begins to rise, indicating that there is no additional benefit of increasing k further (Figure S4A). Thus, in our analysis, a total of 9 PEER factors could account for the observed variance in cell behavior.

We next evaluated whether any PEER factor(s) captured the variance in cell behavior due to the different fibronectin concentrations (Figure 3). The effect of fibronectin was statistically significant (paired t test) for all three concentrations in the case of Factors 1, 3, and 7 (Figure 3). Of those, PEER Factor 1 was the factor that best captured the variance based on statistical analysis. All of the other variance in cell phenotypes attributable to the different fibronectin concentrations (including mean and standard deviation of total number of attached cells, cell and nucleus area and DAPI staining intensity) was also captured by PEER Factor 1 to a greater extent than the other factors (Figure S4B).

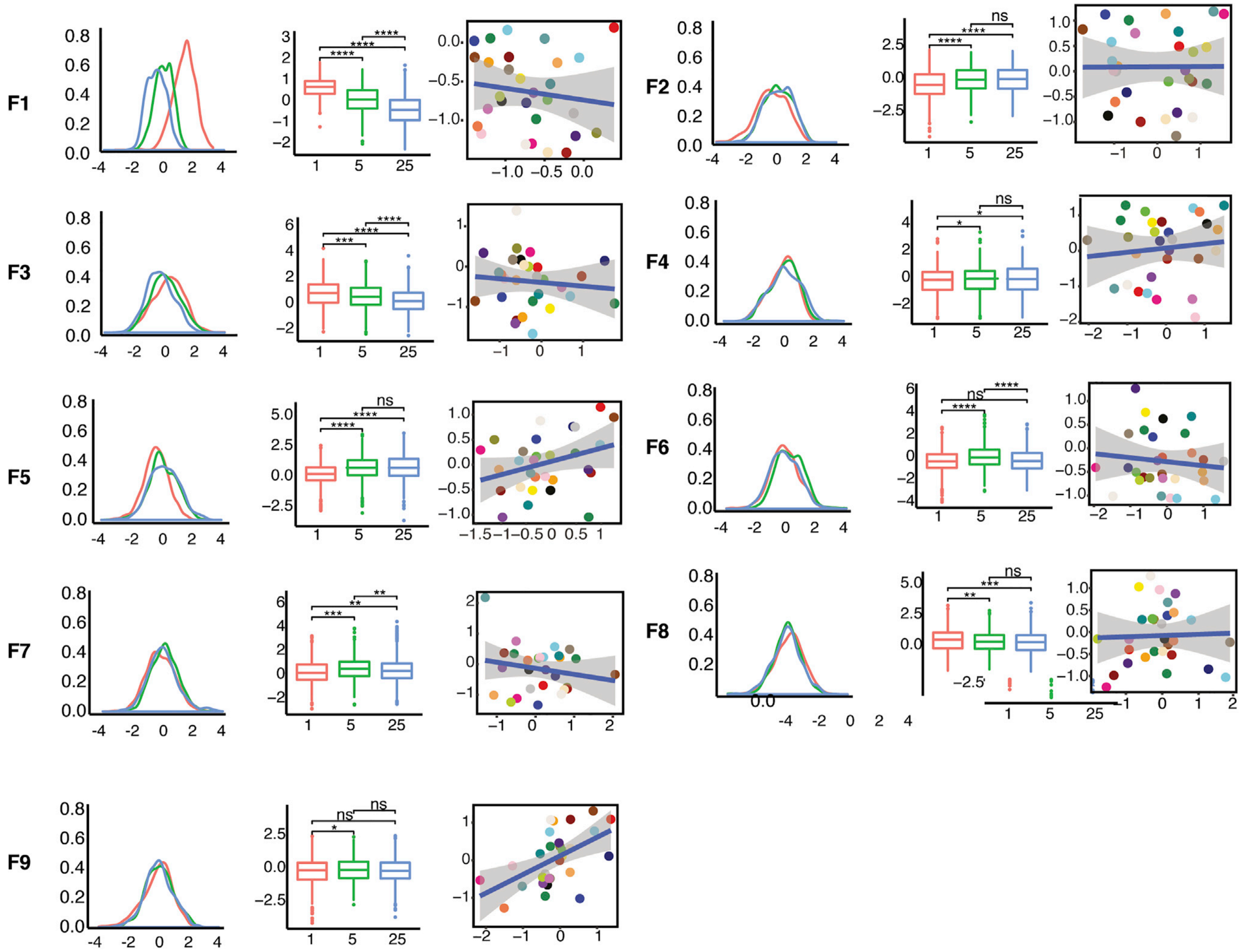


**Figure 3. Synthetic Phenotypic Features Capture Extrinsic and Intrinsic Contributions to Variance**

Plots showing the distribution of values for the 9 PEER Factors (F1–F9). Left and middle columns show distributions for three fibronectin concentrations (Fn1, red; Fn5, green; Fn25, blue). Asterisks represent significance values from pairwise t tests performed between each fibronectin condition (****p $\leq$ 0.0001; ***p $\leq$ 0.001; **p $\leq$ 0.01; *p $\leq$ 0.05; ns, not significant). Right-hand column shows the donor-concordance between two clonal lines of cells derived from the same donors. Values for one cell line in each pair are shown on the x axis and its "twin" on the y axis. Each dot corresponds to one cell line.

We previously reported a donor contribution in the range of 8%–23% to the observed variation in cell behavior (Kilpinen et al., 2017). We therefore hypothesized that one or more PEER factors would capture structure in the data that was dependent on the genetic background of the donors from whom the cell lines were generated. In the cases where we had cell behavior data for two independent lines from the same donor, we plotted phenotypic data for one of the cell lines on the x axis and the other ("twin") cell line on the y axis. Donor concordance is indicated by a positive correlation between the measurements for each pair of lines. This was highest in the case of PEER Factor 9, and therefore this was the factor that best captured intrinsic variance (Figure 3). Phenotypic features describing EdU labeling and other nuclear properties, both in single and clumped cells, loaded onto PEER Factor 9 (Figure S4B). PEER Factor 9 did not capture any of the variation due to FN concentration (Figure 3).

## Identification of Genes Correlating with Extrinsic and Intrinsic Variation

To identify genes whose expression correlated with phenotypic variance, we performed a correlation analysis between the intrinsic (PEER Factor 9) and extrinsic (PEER Factor 1) factors and gene expression array data independently generated from cell pellets as part of the HipSci resource. The gene expression datasets were generated from cell lines between passages 8–41. There was no significant variation in the RNA sequencing (RNA-seq) expression of the majority of genes, including pluripotency factors NANOG and OCT4, with passage number (Kilpinen et al., 2017).

The expression of 4,573 genes correlated with PEER Factor 1 (PEER 1) or PEER 9, or both factors, in at least one fibronectin concentration (Table S3). These genes could be a mixture of genes that are causal, proxy, or a consequence of the cellular trait captured by PEER. From this list, we filtered out genes

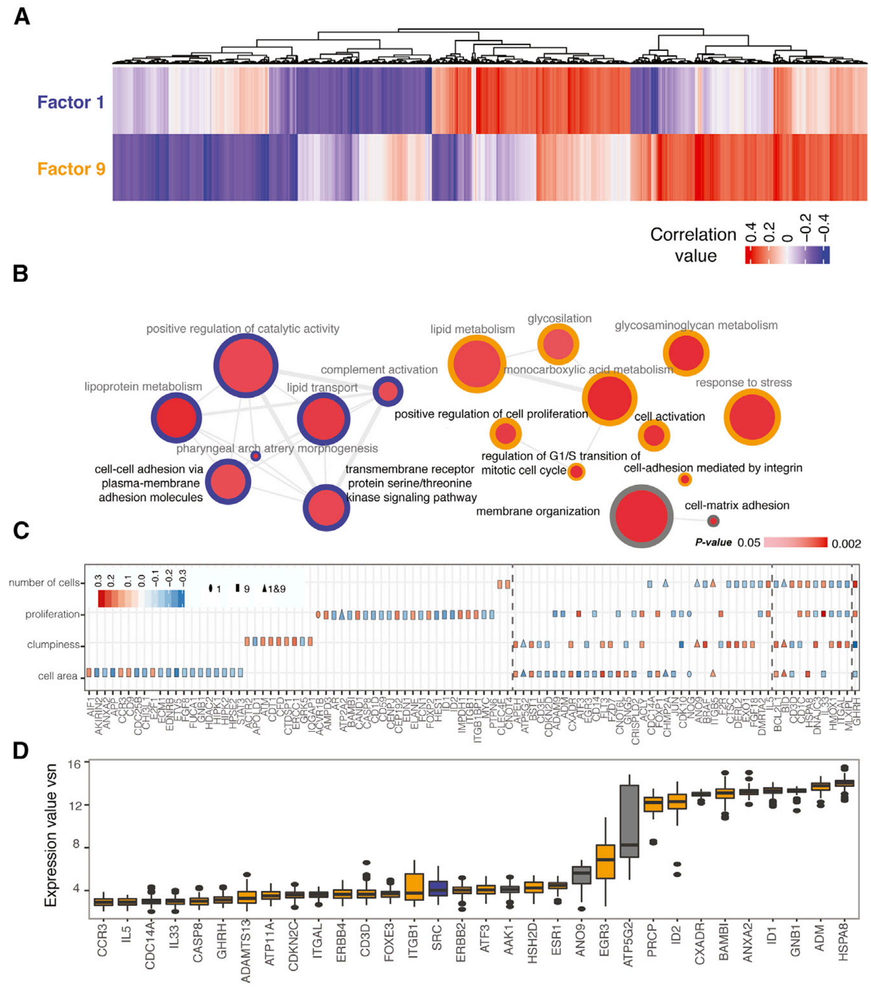


**Figure 4. Using the "Extrinsic" and "Intrinsic" PEER Factors to Identify Genes That Correlate with Specific Cell Phenotypes**

(A) Heatmap showing the 3,879 genes correlated with either extrinsic PEER Factor 1, intrinsic Factor 9, or both. Color scale depicts correlation values.

(B) GO analysis of genes correlating with PEER 1 (blue circles), PEER 9 (orange circles), or both factors (gray circles). All GO terms for the factors are shown. Circle size represents the frequency of the GO term in the underlying Gene Ontology Annotation (GOA) database; red color scale indicates p value. Each gene was mapped to the most specific terms applicable in each ontology. Highly similar GO terms are linked by edges, with edge width depicting the degree of similarity. Terms in black font were used to select the list of 175 genes in Table S3.

(C) In 98 out of 175 genes, gene expression correlated significantly with cell area, tendency to form clumps ("clumpiness"), number of cells, and/or proliferation. The colors of the points correspond to the correlation values, while the shapes indicate correlation of a specific gene to the extrinsic (PEER 1; oval), intrinsic (PEER 9; rectangle) or both (triangles) factors. Grey dotted vertical lines separate genes correlating with one, two or four phenotypes (left to right).

(D) Boxplots showing the expression values (vsn) of 32 out of the 38 genes (Table S5) with outlier gene expression in one or more outlier cell line. Color code (blue, orange, gray) as in (B).

that were not associated with any Ensembl identifiers. We also removed genes for which multiple probes showed opposite correlation values. The resulting dataset consisted of 3,879 genes (Table S3), 1,321 correlating with PEER 1, 1,977 correlating with PEER 9, and 581 with both (Figure 4A).

Gene Ontology (GO) analysis was performed on the 3,879 genes at a threshold value of ±0.2 of the correlation coefficient (Figure 4B; Table S4). All of the GO terms associated with PEER 1 and PEER 9 are shown in Figure 4B. GO terms associated with PEER 1 included cell adhesion and receptor serine and threonine kinase signaling. Terms associated with PEER 9 included cell proliferation, response to stress, and integrin-mediated cell adhesion. Only two GO terms were associated with both PEER factors: membrane organization and cell-matrix adhesion.

Based on the phenotypes measured in our study, we further filtered the genes according to the functions of their protein products. Because we measured EdU incorporation, the relevant GO terms are cell cycle and regulation of cell proliferation. Cell-extracellular matrix adhesion is the relevant GO term to capture adhesion to fibronectin. We also measured adhesion of cells in groups, and therefore cell-cell adhesion is the relevant GO term. The additional GO terms membrane organization and transmembrane receptor signaling are relevant to all the measured phenotypes: proliferation, ECM adhesion, and cell-cell adhesion (Table S2). Within the 3,879 genes correlating with either or both PEER Factors, 175 genes belonging to these 6 GO categories were found. The expression of 98 out of the 175 genes showed a statistically significant correlation with at least 1 of our raw phenotypic features (Table S3). The importance of performing a dimensionality reduction analysis on the phenotypic data and then using the selected factors for the correlation with gene expression data instead of raw phenotypes is confirmed by the quantile (Q-Q) plots in Figure S4C.

Examples of gene expression variation among cell lines for genes correlating with one, two, three, or four phenotypic features (cell number, proliferation, cell clumping, and cell area) are shown in Figure 4C. We noted that most genes showed distinct correlations with the PEER factors (Figure 4C; Table S3). In addition, opposite correlations were found for a given gene and one or more phenotypes. For example, ITGAL, which mediates intercellular adhesion, was positively correlated with clumping and negatively correlated with proliferation.

A total of 38 out of 175 genes showed outlier expression (5$^{th}$ and 95$^{th}$ percentiles) in one or more cell line (Figure 4D). The majority of these genes (32 out of 38) were outliers in outlier cell lines (Table S5). The only outlier gene exclusively associated with

OPEN ACCESS
CellPress

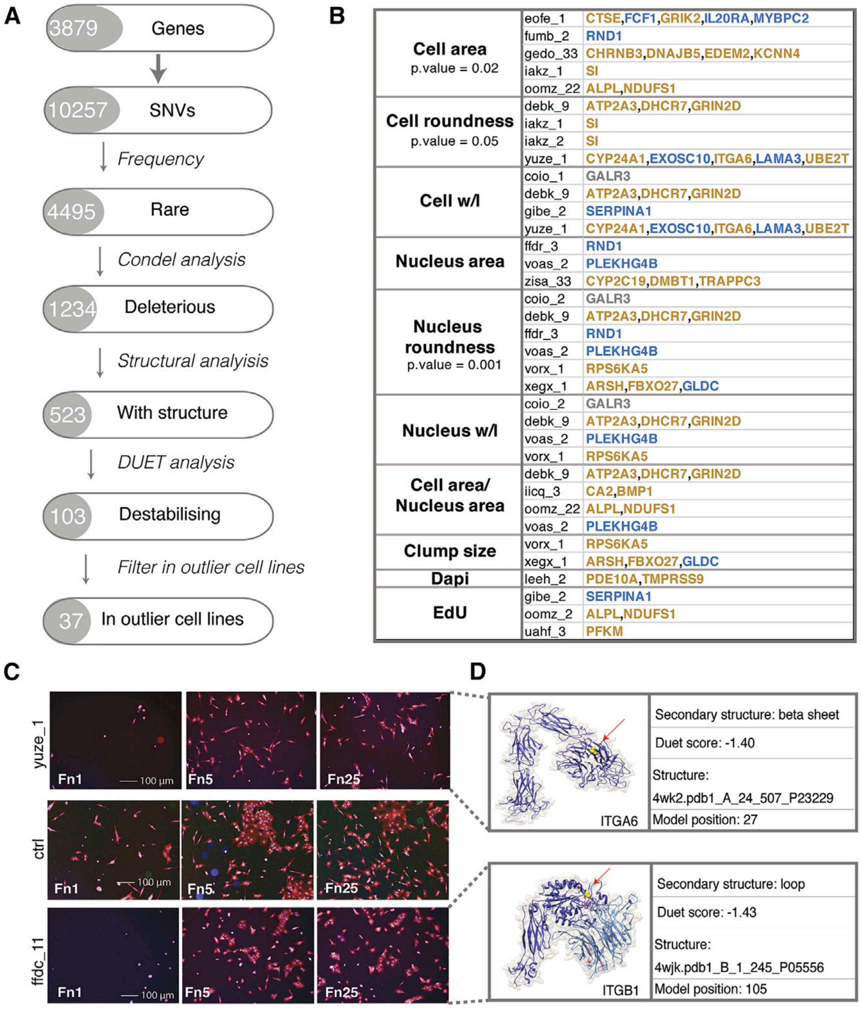


**Figure 5. Identification of Rare, Deleterious, and Destabilizing nsSNVs That Correlate with Outlier Cell Behavior**

(A) Analysis pipeline for selection of genes. The 3,879 genes associated with PEER 1 and 9 were screened for nsSNVs in over 700 cell lines from the HipSci resource and further filtered as shown.

(B) Genes with at least one rare, deleterious, and destabilizing nsSNV in at least one cell line found to be an outlier for one or more phenotype. See Figure 2 for outlier KS analysis. Genes correlating with PEER Factor 1: blue; PEER Factor 9: orange; both: gray. The phenotypes of cell area, cell roundness, and nucleus roundness were significantly over-represented in outlier cell lines with one or more deleterious and destabilizing nsSNV ($p \leq 0.05$).

(C) Representative images of outlier cell line yuze_1 (top), control cell line A1ATD-iPSC patient 1 (center), and cell line not analyzed in the original screen ffdc_11 (bottom), on different fibronectin concentrations (Fn1, Fn5, and Fn25).

(D) Protein structures of integrin α6 (top) and integrin β1 (bottom). nsSNVs detected in the two cell lines are shown with yellow spots indicated by red arrows.

PEER 9 was SRC, proto-oncogene tyrosine-protein kinase. However, in cases in which two cell lines from the same donor were outliers for the same raw phenotypic features (Figure 2), this did not correlate with an overexpression or lack of expression of the same set of genes.

In conclusion, we could identify a large number of mRNAs that correlated with modal cell behavior and a smaller number that correlated with outlier behavior. The GO terms were, for the most part, those that would be predicted to be associated with the types of phenotypic measurement that we recorded.

## Identification of nsSNVs in Cell Adhesion Genes that Correlate with Outlier Cell Phenotypes

Because most of the mRNAs loading onto PEER 1 and PEER 9 correlated with modal, rather than outlier cell behavior, we explored the alternative hypothesis that the presence of single nucleotide variants (nsSNVs) in gene exons that affected protein function would correlate with outlier cell behavior. We searched all the cell lines in the Hipsci resource (>700 lines) for nsSNVs in the 3,879 genes (Table S3) identified with the extrinsic and intrinsic PEER factors (Figure 5A; Table S6). Of the 10,257 nsSNVs identified, 4,495 were classified as rare, based first on the 1000 Genomes Project Consortium (2015) and ExAC (Lek et al., 2016) and second on the frequency in our cell lines (present in fewer than 5 out of 110 lines) (Figure 5A). We further filtered the nsSNVs by using the computational model DUET (Pires et al., 2014) to predict nsSNVs that would be deleterious and the computational model Condel (González-Pérez and López-Bigas, 2011) to predict a final list of 103 rare, deleterious, and destabilizing nsSNVs that would impair protein structure. Among the genes that we identified (Table S6), several encoded proteins were associated with cell adhesion, including integrins and cytoskeleton and ECM proteins.

A total of 37 of the 103 rare, deleterious, and destabilizing nsSNVs occurred in cell lines that were outliers for one or more phenotype (Figure 5B; Table S6). In Figure 5B, those genes associated with PEER Factor 1 are marked in blue, those associated with PEER Factor 9 in orange, and the gene associated with both in gray. The phenotypes of cell area, cell roundness, and nucleus roundness were significantly over-represented in outlier cell lines with one or more deleterious and destabilizing nsSNV (Fisher's exact test comparing the number of outlier cell lines with and without nsSNVs against non-outlier cell lines) (Figure 5B).

Integrins are heterodimeric proteins that mediate binding to fibronectin and other extracellular matrix proteins. We identified a nsSNV in ITGA6, encoding the α6 integrin subunit, in yuze_1, which maps to the integrin ligand-binding domain (Figures 5B–5D). Consistent with the predicted deleterious effect of this mutation, Yuze_1 is an outlier for cell roundness and width-to-length ratio (Figure 5B) and shows reduced spreading, particularly on

the lowest concentration of fibronectin (Figure 2; Figure 5C). We also found a nsSNV in the ligand-binding domain of ITGB1, encoding the β1 integrin subunit, in one of the HipSci cell lines, ffdc_11, that had not been included in the phenotypic screen. When plated on fibronectin, the ffdc_11 line also exhibited reduced attachment and spreading on the lowest fibronectin concentration (Figure 5C), indicative of the predicted outlier phenotype. Thus, we were able to predict outlier cell behavior based on a nsSNV in an integrin gene.

## DISCUSSION

Genetic mapping provides an unbiased approach to discovering genes that influence disease traits and responses to environmental stimuli, such as drug exposure (McCarthy et al., 2008). The attractions of developing human *in vitro* models that reflect *in vivo* genetics and physiology for mechanistic studies are obvious and include quantitation by high-content image analysis and the replacement of animal experiments. The concept that human-disease-causing mutations result in alterations in cell behavior that can be detected in culture is well established, as in the case of keratin mutations affecting the properties of cultured epidermal cells (Knöbel et al., 2015). In addition, human lymphoblastoid cell lines have long been used to model genotype-phenotype relationships in healthy individuals, although limitations include the confounding effects of biological noise and differentiation state, and variation in passage number and proliferation rate (Choy et al., 2008; Jack et al., 2014).

There has been renewed interest in applying human iPSCs for pharmacogenomics, disease modeling, and uncovering genetic modifiers of complex disease traits (Barral and Kurian, 2016). For example, studies with iPSC-derived neurons (Brennand and Gage, 2011) support the "watershed model" (Cannon and Keller, 2006), whereby many different combinations of malfunctioning genes disrupt a few essential pathways to result in the disease. For these reasons, we decided to extend the iPSC approach in an attempt to identify genetic modifiers of cell behavior in healthy individuals. We have recently reported that in an analysis of over 700 well-characterized human iPSC lines, there is an 8%–23% genetic contribution to variation in cell behavior (Kilpinen et al., 2017). Our ability to detect this contribution depended on the use of simple, short-term, quantitative assays of cell behavior; the application of multiple environmental stimuli (different concentrations of fibronectin; single cells versus cell clumps); and homogeneous starting cell populations for the assays. The concept that genetic background contributes to the variability of human iPSCs is supported by a number of earlier studies (Kyttälä et al., 2016; Burrows et al., 2016; Rouhani et al., 2014).

In order to explore the nature of the genetic contribution to variation in cell behavior, we developed computational approaches to integrate genomic, gene expression, and cell biology datasets. Previously, we had taken a GWAS approach (Kilpinen et al., 2017) and found only 6 variants where the lead expression quantitative trait locus (eQTL) variant was identical to a cataloged GWAS variant, including an eQTL variant for the *TERT* gene. This was one of our motivations for developing different approaches. We applied a dimensionality reduction approach, PEER, to capture variance due to extrinsic contributors (different fibronectin concentrations) and genetic concordance. This revealed a robust correlation between RNA expression and the phenotypic features in a large panel of iPSC lines, with the expression of specific RNAs associated with intrinsic or extrinsic factors. Carcamo-Orive et al. (2017) also found that human iPSC lines retain a donor-specific gene expression pattern. However, in that study, cells were not exposed to different environmental stimuli.

The majority of human iPSCs we screened responded in the same way to a given FN concentration. This likely reflects canalisation, the process by which normal organs and tissues are produced even on a background of slight genetic abnormalities (Rutherford and Lindquist, 1998; McLaren, 1999). However, we did identify cell lines that exhibited outlier behavior that could not be accounted for by variation in gene expression levels (see Figure 4), leading us to hypothesize that outlier phenotypes might correlate with genetic variants. We identified rare nsSNVs that were predicted to be deleterious and for which protein structural information was available. Some of the nsSNVs identified by this approach occurred in cell lines that were outliers for one or more phenotypes, such as cell spreading. The phenotypes of cell area, cell roundness, and nucleus roundness were significantly over-represented in outlier cell lines with one or more deleterious and destabilizing nsSNV. The identification of nsSNVs in integrin genes is of particular interest, because integrins are highly polymorphic and some of the previously reported nsSNVs alter adhesive functions of cancer cells (Ferreira et al., 2009; Evans et al., 2003).

In conclusion, our platform has been successful in associating specific RNAs with intrinsic or extrinsic factors and discovering nsSNVs that correlate with outlier cell behavior. This represents a major advance in attempts to map genetic variation to phenotypic variation.

## STAR★METHODS

Detailed methods are provided in the online version of this paper and include the following:

- KEY RESOURCES TABLE
- CONTACT FOR REAGENT AND RESOURCE SHARING
- EXPERIMENTAL MODEL AND SUBJECT DETAILS
    - ○ Cell line derivation and culture
    - ○ Mycoplasma testing and STR profiling
- METHOD DETAILS
    - ○ Fibronectin adhesion assays
    - ○ Gene expression profiling
    - ○ Dimensionality reduction approach
    - ○ Gene Ontology analysis
    - ○ Single Nucleotide Variation (SNV) analysis
- QUANTIFICATION AND STATISTICAL ANALYSIS
- DATA AND SOFTWARE AVAILABILITY

## SUPPLEMENTAL INFORMATION

Supplemental Information can be found with this article online at https://doi.org/10.1016/j.celrep.2019.01.094.

## CONSORTIA

The members of HipSci can be viewed at https://journals.plos.org/plosone/article/file?id=10.1371/journal.pone.0155014.s003&type=supplementary.

## ACKNOWLEDGMENTS

We are grateful to the Wellcome Trust and MRC for funding through the Human Induced Pluripotent Stem Cell Initiative (WT098503). We also gratefully acknowledge funding from the Department of Health by the National Institute for Health Research comprehensive Biomedical Research Centre award to Guy's & St Thomas' National Health Service Foundation Trust in partnership with King's College London and King's College Hospital NHS Foundation Trust. The views expressed are those of the author(s) and not necessarily those of the NHS, the NIHR or the Department of Health. A.V. and N.M.L. gratefully acknowledge the support of The Francis Crick Institute, which receives its core funding from Cancer Research UK (FC001110), the UK Medical Research Council (FC001110), and the Wellcome Trust (FC001110). We thank members of the Centre for Stem Cells and Regenerative Medicine for scientific discussions and Mia Gervasio, Sabrina Munir, Ayaulim Nurgozhina, Fatima Chowdhury, Darrick Hansen, Zuming Tang, Christopher Sibley-Allen, and Fran Molina for technical support. F.M.W. is grateful to Mark Walport for making this research possible.

## AUTHOR CONTRIBUTIONS

F.M.W.: conceptualization; A.V., A. Leha, A. Laddach, A.G., E.W., F.F., and O.S.: formal analysis; N.M., R.M., O.J.C., A.K., C.H., A.V., M.T., and D.D.: investigation; E.B., P.W.Z., and HipSci consortium: resources; A.V., N.M.L., D.D., and F.M.W.: writing; and R.D. and F.M.W.: funding acquisition.

## DECLARATION OF INTERESTS

F.M.W. is currently on secondment as Executive Chair, Medical Research Council.

Received: June 18, 2018
Revised: December 11, 2018
Accepted: January 24, 2019
Published: February 19, 2019

## REFERENCES

Altschul, S.F., Madden, T.L., Schäffer, A.A., Zhang, J., Zhang, Z., Miller, W., and Lipman, D.J. (1997). Gapped BLAST and PSI-BLAST: a new generation of protein database search programs. Nucleic Acids Res. *25*, 3389–3402.

1000 Genomes Project Consortium; Auton, A., Brooks, L.D., Durbin, R.M., Garrison, E.P., Kang, H.M., Korbel, J.O., Marchini, J.L., McCarthy, S., McVean, G.A., and Abecasis, G.R. (2015). A global reference for human genetic variation. Nature *526*, 68–74.

Barral, S., and Kurian, M.A. (2016). Utility of induced pluripotent stem cells for the study and treatment of genetic diseases: focus on childhood neurological disorders. Front. Mol. Neurosci. *9*, 78.

Berman, H., Henrick, K., and Nakamura, H. (2003). Announcing the worldwide Protein Data Bank. Nat. Struct. Biol. *10*, 980.

Brennand, K.J., and Gage, F.H. (2011). Concise review: the promise of human induced pluripotent stem cell-based studies of schizophrenia. Stem Cells *29*, 1915–1922.

Burrows, C.K., Banovich, N.E., Pavlovic, B.J., Patterson, K., Gallego Romero, I., Pritchard, J.K., and Gilad, Y. (2016). Genetic variation, not cell type of origin, underlies the majority of identifiable regulatory differences in iPSCs. PLoS Genet. *12*, e1005793.

Cannon, T.D., and Keller, M.C. (2006). Endophenotypes in the genetic analyses of mental disorders. Annu. Rev. Clin. Psychol. *2*, 267–290.

Carcamo-Orive, I., Hoffman, G.E., Cundiff, P., Beckmann, N.D., D'Souza, S.L., Knowles, J.W., Patel, A., Papatsenko, D., Abbasi, F., Reaven, G.M., et al. (2017). Analysis of transcriptional variability in a large human iPSC library reveals genetic and non-genetic determinants of heterogeneity. Cell Stem Cell *20*, 518–532.e9.

Choi, J., Goh, G., Walradt, T., Hong, B.S., Bunick, C.G., Chen, K., Bjornson, R.D., Maman, Y., Wang, T., Tordoff, J., et al. (2015). Genomic landscape of cutaneous T cell lymphoma. Nat. Genet. *47*, 1011–1019.

Choy, E., Yelensky, R., Bonakdar, S., Plenge, R.M., Saxena, R., De Jager, P.L., Shaw, S.Y., Wolfish, C.S., Slavik, J.M., Cotsapas, C., et al. (2008). Genetic analysis of human traits in vitro: drug response and gene expression in lymphoblastoid cell lines. PLoS Genet. *4*, e1000287.

DeBoever, C., Li, H., Jakubosky, D., Benaglio, P., Reyna, J., Olson, K.M., Huang, H., Biggs, W., Sandoval, E., D'Antonio, M., et al. (2017). Large-scale profiling reveals the influence of genetic variation on gene expression in human induced pluripotent stem cells. Cell Stem Cell *20*, 533–546.e7.

Evans, R.D., Perkins, V.C., Henry, A., Stephens, P.E., Robinson, M.K., and Watt, F.M. (2003). A tumor-associated beta 1 integrin mutation that abrogates epithelial differentiation control. J. Cell Biol. *160*, 589–596.

Ferreira, M., Fujiwara, H., Morita, K., and Watt, F.M. (2009). An activating beta1 integrin mutation increases the conversion of benign to malignant skin tumors. Cancer Res. *69*, 1334–1342.

Finn, R.D., Clements, J., and Eddy, S.R. (2011). HMMER web server: interactive sequence similarity searching. Nucleic Acids Res. *39*, W29–37.

Finn, R.D., Coggill, P., Eberhardt, R.Y., Eddy, S.R., Mistry, J., Mitchell, A.L., Potter, S.C., Punta, M., Qureshi, M., Sangrador-Vegas, A., et al. (2016). The Pfam protein families database: towards a more sustainable future. Nucleic Acids Res. *44* (*D1*), D279–D285.

González-Pérez, A., and López-Bigas, N. (2011). Improving the assessment of the outcome of nonsynonymous SNVs with a consensus deleteriousness score, Condel. Am. J. Hum. Genet. *88*, 440–449.

Huber, W., von Heydebreck, A., Sültmann, H., Poustka, A., and Vingron, M. (2002). Variance stabilization applied to microarray data calibration and to the quantification of differential expression. Bioinformatics *18* (*Suppl 1*), S96–S104.

Jack, J., Rotroff, D., and Motsinger-Reif, A. (2014). Lymphoblastoid cell lines models of drug response: successes and lessons from this pharmacogenomic model. Curr. Mol. Med. *14*, 833–840.

Kang, H.M., Ye, C., and Eskin, E. (2008). Accurate discovery of expression quantitative trait loci under confounding from spurious and genuine regulatory hotspots. Genetics *180*, 1909–1925.

Kilpinen, H., Goncalves, A., Leha, A., Afzal, V., Alasoo, K., Ashford, S., Bala, S., Bensaddek, D., Casale, F.P., Culley, O.J., et al. (2017). Common genetic variation drives molecular heterogeneity in human iPSCs. Nature *546*, 370–375.

Knöbel, M., O'Toole, E.A., and Smith, F.J. (2015). Keratins and skin disease. Cell Tissue Res. *360*, 583–589.

Kyttälä, A., Moraghebi, R., Valensisi, C., Kettunen, J., Andrus, C., Pasumarthy, K.K., Nakanishi, M., Nishimura, K., Ohtaka, M., Weltner, J., et al. (2016). Genetic variability overrides the impact of parental cell type and determines iPSC differentiation potential. Stem Cell Reports *6*, 200–212.

Lane, S.W., Williams, D.A., and Watt, F.M. (2014). Modulating the stem cell niche for tissue regeneration. Nat. Biotechnol. *32*, 795–803.

Laurent, L.C., Ulitsky, I., Slavin, I., Tran, H., Schork, A., Morey, R., Lynch, C., Harness, J.V., Lee, S., Barrero, M.J., et al. (2011). Dynamic changes in the copy number of pluripotency and cell proliferation genes in human ESCs and iPSCs during reprogramming and time in culture. Cell Stem Cell *8*, 106–118.

Leek, J.T., and Storey, J.D. (2007). Capturing heterogeneity in gene expression studies by surrogate variable analysis. PLoS Genet. *3*, 1724–1735.

Leha, A., Moens, N., Meleckyte, R., Culley, O.J., Gervasio, M.K., Kerz, M., Reimer, A., Cain, S.A., Streeter, I., Folarin, A., et al.; HipSci Consortium (2016). A high-content platform to characterise human induced pluripotent stem cell lines. Methods *96*, 85–96.

Lek, M., Karczewski, K.J., Minikel, E.V., Samocha, K.E., Banks, E., Fennell, T., O'Donnell-Luria, A.H., Ware, J.S., Hill, A.J., Cummings, B.B., et al.; Exome Aggregation Consortium (2016). Analysis of protein-coding genetic variation in 60,706 humans. Nature *536*, 285–291.

McCarthy, M.I., Abecasis, G.R., Cardon, L.R., Goldstein, D.B., Little, J., Ioannidis, J.P., and Hirschhorn, J.N. (2008). Genome-wide association studies for complex traits: consensus, uncertainty and challenges. Nat. Rev. Genet. *9*, 356–369.

McLaren, A. (1999). Signaling for germ cells. Genes Dev. *13*, 373–376.

Müller, F.J., Schuldt, B.M., Williams, R., Mason, D., Altun, G., Papapetrou, E.P., Danner, S., Goldmann, J.E., Herbst, A., Schmidt, N.O., et al. (2011). A bioinformatic assay for pluripotency in human cells. Nat. Methods *8*, 315–317.

Notredame, C., Higgins, D.G., and Heringa, J. (2000). T-Coffee: a novel method for fast and accurate multiple sequence alignment. J. Mol. Biol. *302*, 205–217.

Panopoulos, A.D., D'Antonio, M., Benaglio, P., Williams, R., Hashem, S.I., Schuldt, B.M., DeBoever, C., Arias, A.D., Garcia, M., Nelson, B.C., et al. (2017). IPSCORE: a resource of 222 iPSC lines enabling functional characterization of genetic variation across a variety of cell types. Stem Cell Reports *8*, 1086–1100.

Pashos, E.E., Park, Y., Wang, X., Raghavan, A., Yang, W., Abbey, D., Peters, D.T., Arbelaez, J., Hernandez, M., Kuperwasser, N., et al. (2017). Large, diverse population cohorts of hiPSCs and derived hepatocyte-like cells reveal functional genetic variation at blood lipid-associated loci. Cell Stem Cell *20*, 558–570.e10.

Pires, D.E.V., Ascher, D.B., and Blundell, T.L. (2014). DUET: a server for predicting effects of mutations on protein stability using an integrated computational approach. Nucleic Acids Res. *42*, W314–9.

Rashid, S.T., Corbineau, S., Hannan, N., Marciniak, S.J., Miranda, E., Alexander, G., Huang-Doran, I., Griffin, J., Ahrlund-Richter, L., Skepper, J., et al. (2010). Modeling inherited metabolic disorders of the liver using human induced pluripotent stem cells. J. Clin. Invest. *120*, 3127–3136.

Reimer, A., Vasilevich, A., Hulshof, F., Viswanathan, P., van Blitterswijk, C.A., de Boer, J., and Watt, F.M. (2016). Scalable topographies to support proliferation and Oct4 expression by human induced pluripotent stem cells. Sci. Rep. *6*, 18948.

Robinton, D.A., and Daley, G.Q. (2012). The promise of induced pluripotent stem cells in research and therapy. Nature *481*, 295–305.

Rouhani, F., Kumasaka, N., de Brito, M.C., Bradley, A., Vallier, L., and Gaffney, D. (2014). Genetic background drives transcriptional variation in human induced pluripotent stem cells. PLoS Genet. *10*, e1004432.

Rutherford, S.L., and Lindquist, S. (1998). Hsp90 as a capacitor for morphological evolution. Nature *396*, 336–342.

Shen, M.Y., and Sali, A. (2006). Statistical potential for assessment and prediction of protein structures. Protein Sci. *15*, 2507–2524.

Stegle, O., Kannan, A., Durbin, R., and Winn, J. (2008). Accounting for non-genetic factors improves the power of eQTL studies. In Research in Computational Molecular Biology. RECOMB 2008, M. Vingron and L. Wong, eds. (Springer), pp. 411–422.

Stegle, O., Parts, L., Piipari, M., Winn, J., and Durbin, R. (2012). Using probabilistic estimation of expression residuals (PEER) to obtain increased power and interpretability of gene expression analyses. Nat. Protoc. *7*, 500–507.

The UniProt Consortium. (2017). UniProt: the universal protein knowledgebase. Nucleic Acids Res. *45* (*D1*), D158–D169.

Wang, K., Li, M., and Hakonarson, H. (2010). ANNOVAR: functional annotation of genetic variants from high-throughput sequencing data. Nucleic Acids Res. *38*, e164.

Warren, C.R., O'Sullivan, J.F., Friesen, M., Becker, C.E., Zhang, X., Liu, P., Wakabayashi, Y., Morningstar, J.E., Shi, X., Choi, J., et al. (2017). Induced pluripotent stem cell differentiation enables functional validation of GWAS variants in metabolic disease. Cell Stem Cell *20*, 547–557.e7.

Webb, B., and Sali, A. (2016). Comparative protein structure modeling using MODELLER. Curr. Protoc. Bioinformatics *54*, 5.6.1–5.6.37.

# STAR★METHODS

## KEY RESOURCES TABLE

| REAGENT or RESOURCE | SOURCE | IDENTIFIER |
|---|---|---|
| Antibodies | | |
| Oct4 | Santa Cruz | Sc5279 |
| NANOG | Abcam | Ab80892 |
| Chemicals, Peptides, and Recombinant Proteins | | |
| Human plasma fibronectin | Corning | 356008 |
| ROCK inhibitor | Enzo | Y-27632 |
| Accutase | Biolegend | 423201 |
| Click-iT EdU kit | Life Technologies | C10337 |
| CellMask | Life Technologies | *C10046* |
| DAPI | ThermoFisher | D1306 |
| Deposited Data | | |
| Gene expression | https://www.ebi.ac.uk/arrayexpress/experiments/E-MTAB-4057/ | N/A |
| Image data resource | https://idr.openmicroscopy.org/webclient/?show=screen-2051 | Idr0037 |
| Experimental Models: Cell Lines | | |
| Human iPSCs | https://www.sanger.ac.uk/science/collaboration/hipsci | Table S1 |
| Oligonucleotides | | |
| 5′GGGAGCAAACAGGATTAGATACCCT3′ | Sigma | Mycoplasma FW |
| 5′TGCACCATCTGTCACTCTGTTAACCTC3′ | Sigma | Mycoplasma Rev |
| Probe Hs04260367_gH Taqman | ThermoFisher | Oct4 |
| Software and Algorithms | | |
| Harmony v4.1 software | https://support.myharmony.com/en-gb/ | Perkin Elmer |
| Gene Ontology | http://cbl-gorilla.cs.technion.ac.il/ | N/A |

## CONTACT FOR REAGENT AND RESOURCE SHARING

Further information and requests for resources and reagents should be directed to and will be fulfilled by the Lead Contact, Fiona Watt (fiona.watt@kcl.ac.uk).

## EXPERIMENTAL MODEL AND SUBJECT DETAILS

### Cell line derivation and culture

All HipSci samples were collected from consented research volunteers recruited from the NIHR Cambridge BioResource (https://www.cambridgebioresource.group.cam.ac.uk/). Human iPSC were generated from fibroblasts by transduction with Sendai vectors expressing hOCT3/4, hSOX2, hKLF4, and hc-MYC (CytoTune, Life Technologies, Cat. no. A1377801). Cells were cultured on irradiated or Mitomycin C-treated mouse embryonic fibroblasts (MEF-CF1) in advanced DMEM (Life technologies, UK) supplemented with 10% Knockout Serum Replacement (KOSR, Life technologies, UK), 2 mM L-glutamine (Life technologies, UK) 0.007% 2-mercaptoethanol (Sigma-Aldrich, UK), 4 ng/mL recombinant Fibroblast Growth Factor-2, and 1% Pen/Strep (Life technologies, UK). Pluripotency was assessed based on expression profiling (Müller et al., 2011), detection of pluripotency markers in culture and response to differentiation inducing conditions (Robinton and Daley, 2012). Established iPSC lines were passaged every 3-4 days approximately at a 1:3 split ratio. The ID numbers and details for each cell line are listed in Table S1.

### Mycoplasma testing and STR profiling

For mycoplasma testing 1 mL of conditioned medium was heated for 5min at 95°C. A PCR reaction was set up with the following primers: forward (5′GGGAGCAAACAGGATTAGATACCCT3′); reverse (5′TGCACCATCTGTCACTCTGTTAACCTC3′). PCR products were loaded on a 1% w/v agarose gel, run at 110 V for 30 minutes in TAE buffer and observed with a Gel Dox XR+ imaging system (Bio-Rad). To confirm cell line identity, DNA extraction was performed using the DNeasy Blood & Tissue Kit (QIAGEN). Confluent cells were dissociated from 6-well plates and lysed in protein K solution; 4 μL of 100mg/ml RNase solution (QIAGEN) was added and DNA

was purified through the spin-column and eluted in 150 μl. DNA quality was confirmed with a nanodrop spectrophotometer (Nanodrop 2000, Thermo scientific) and on a 1% agarose gel. DNA samples were sequenced using STR profiling at the Wellcome Trust Sanger Institute.

## METHOD DETAILS

### Fibronectin adhesion assays

96-well micro-clear-black tissue culture plates (Greiner cat. No. 655090) were coated with three concentrations of human plasma fibronectin (Corning) in alternating columns in a randomized fashion (Leha et al., 2016). Cells were incubated for 8 min with Accutase (Biolegend) to create a single cell suspension. As the cells began to separate and round up, pre-warmed medium containing 10 μM Rho- associated protein kinase (ROCK) inhibitor (Y-27632; Enzo Life Sciences) was added and cells were removed from culture wells by gentle pipetting to form a single cell suspension. Cells were then collected by centrifugation, aspirated and resuspended in medium containing 10 μM ROCK inhibitor. Cells were counted using a Scepter 2.0 automated cell- counting device (Millipore) and seeded onto the fibronectin- coated 96-well plates using Viaflo (INTEGRA Biosciences) electronic pipettes.

Cell line plating was randomized within rows, with three wells per condition for each line to obviate edge and position effects. One control line (A1ATD-iPSC patient 1) (Rashid et al., 2010), kindly provided by S. Tamir Rashid and Ludovic Vallier, was run as an internal control in the majority of plates. For each well, 3,000 cells were plated for 24 hours prior to fixation. Paraformaldehyde 8% (PFA, Sigma–Aldrich) was added to an equal volume of medium for a final concentration of 4%, and left at room temperature for 15 min. Cells were labeled with EdU (Click-iT, Life Technologies) 30 minutes before fixation. Fixed cells were blocked and permeabilised with 0.1% v/v Triton X-100 (Sigma–Aldrich), 1% w/v bovine serum albumin (BSA, Sigma–Aldrich) and 3%v/v donkey serum (Sigma–Aldrich) for 20 min at room temperature and stained with DAPI (1 microM final concentration, Life Technologies) and cell mask (1:1000, Life Technologies). EdU was detected according to the manufacturer's instructions, except that the concentration of the azide reagent was reduced by 50%.

Images were acquired using an Operetta (Perkin Elmer) high content device. Border wells were avoided to reduce edge effects. Harmony v4.1 software was used to derive measurements for each cell. Measurements included intensity features (DAPI, EdU), morphology features (cell area, cell roundness, cell width to length ratio, nucleus area, nucleus roundness, nucleus width to length ratio) and context features related to cell adhesion properties (number of cells per clump). Processing quantification and normalization of data were performed as previously described (Leha et al., 2016).

### Gene expression profiling

Gene expression profiles were measured with Illumina HumanHT-12 v4 Expression BeadChips and processed as described by Kilpinen et al., (2017). Probe intensity estimates were normalized separately for the two cell types using the variance-stabilizing transformation implemented in the R/Bioconductor vsn package (Huber et al., 2002). After normalization, the datasets were limited to the final remapped set of probes (n probes = 25,604). We refer to this version of the gexarray data as vsn log2 (iPSC/somatic). PEER analysis was performed taking as input the vsn expression values with the following parameters: K = 36; covariates = cell line and batch; maximum iterations = 10,000. The residual gene expression matrix was used to perform a correlation analysis with both intrinsic/extrinsic factors and raw phenotypes using cor() function in R (method Spearman's).

### Dimensionality reduction approach

We applied a Bayesian factor analysis model called PEER (Stegle et al., 2012) to the phenotype data in each cell line. This approach uses an unsupervised linear model to account for global variance components in the data, and yields a number of factor components that can be used as synthetic phenotypes in further analysis. We tested a wide range of parameter settings for the model (the *k* number), controlling the amount of variance explained by it. We ran PEER with the full pre-normalized dataset with the following parameters: K = 9; covariates = cell line, fibronectin and batch; maximum iterations = 10,000.

### Gene Ontology analysis

Gene Ontology analysis was performed using the Gorilla web-service (http://cbl-gorilla.cs.technion.ac.il/) and the output was visualized with ReviGO (http://revigo.irb.hr/). Three analyses were performed separately for the genes correlating with the extrinsic factor, the intrinsic factor and both factors.

### Single Nucleotide Variation (SNV) analysis

All nsSNVs identified from the "INFO_04_filtered" VCF files from the latest release of the exomeseq data, which have been filtered for higher confidence variants using Impute2, were mapped to protein sequences using ANNOVAR (Wang et al., 2010). Those nsSNVs that mapped to genes in our set of genes were selected for further analysis.

Rare nsSNVs were defined as those with a minor allele frequency (MAF) < 0.005 in both the 1000 Genomes Project (1000 Genomes Project Consortium, 2015) and ExAC database (Lek et al., 2016). Protein domain boundaries were obtained by scanning UNIPROT (The UniProt Consortium, 2017) protein sequences against the PFAM (Finn et al., 2016) seed libraries using HMMER (Finn et al., 2011). UniProt proteins (with mapped nsSNVs) were assigned resolved protein structures/homologs from the PDB biounit database

(Berman et al., 2003) using BLAST (Altschul et al., 1997). BLAST searches were carried out using both the entire protein sequences and domain sequences.

For each protein with mapped nsSNVs the structural homolog with the highest identity was chosen as a template for homology modeling. In the case of ties the modeling process was performed using each template. The portion of the template and query sequences relating to a BLAST hit were aligned using T-COFFEE (Notredame et al., 2000). 10 homology models for each query template alignment were created using the MODELER software (Webb and Sali, 2016). In each case the model with the lowest zDOPE score (Shen and Sali, 2006) was selected for further analysis. Where models were created using several templates the model with the lowest zDOPE out of all created models was selected for further analysis.

The impact of all nsSNVs was assessed using a primarily sequence-based consensus predictor of deleteriousness, Condel (González-Pérez and López-Bigas, 2011). Where structural information was available, the impact of nsSNVs on protein structural stability was also predicted using DUET (Pires et al., 2014).

## QUANTIFICATION AND STATISTICAL ANALYSIS

Quantification and normalization of cell phenotype data were performed as previously described (Leha et al., 2016). For gene expression analysis a p value threshold $< 0.05$ was applied to select the statistically significant correlations and the cut-off of the correlation values was set to $\pm 0.2$. Hits in the BLAST SNV analysis were accepted with a sequence identity $> 30\%$ and E-value $< 0.001$.

## DATA AND SOFTWARE AVAILABILITY

New cell phenotype data have been deposited in an online database in conjunction with the research reported in this paper. The raw image cell phenotypes data are available in the Image Data Resource idr0037 https://idr.openmicroscopy.org/webclient/?show=screen-2051 following on from the previous dataset https://doi.org/10.17867/10000107. Open access gene expression array data are available in the ArrayExpress database (https://www.ebi.ac.uk/arrayexpress/) under accession number https://www.ebi.ac.uk/arrayexpress/experiments/E-MTAB-4057/. The Gorilla web-service for Gene Ontology analysis is available at http://cbl-gorilla.cs.technion.ac.il/ and the ReviGO visualization tool is available at http://revigo.irb.hr/.

Cell Reports, Volume *26*

## Supplemental Information

# Identifying Extrinsic versus Intrinsic Drivers of Variation in Cell Behavior in Human iPSC Lines from Healthy Donors

**Alessandra Vigilante, Anna Laddach, Nathalie Moens, Ruta Meleckyte, Andreas Leha, Arsham Ghahramani, Oliver J. Culley, Annie Kathuria, Chloe Hurling, Alice Vickers, Erika Wiseman, Mukul Tewary, Peter W. Zandstra, HipSci Consortium, Richard Durbin, Franca Fraternali, Oliver Stegle, Ewan Birney, Nicholas M. Luscombe, Davide Danovi, and Fiona M. Watt**

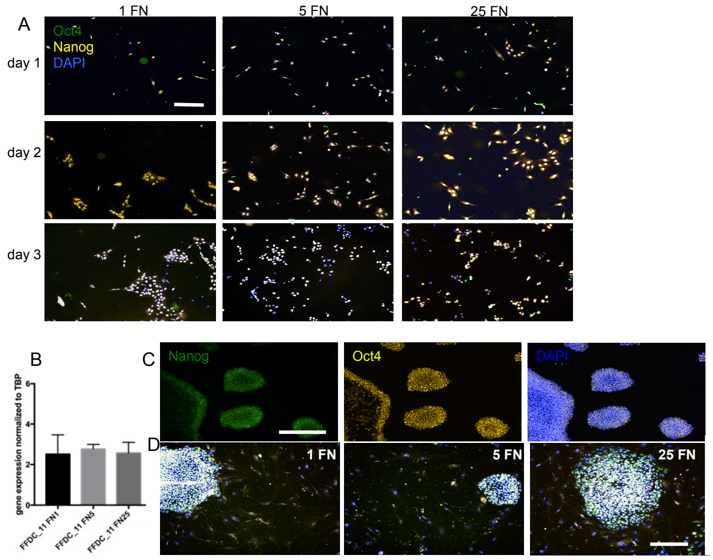


**Figure S1. Related to Figure 1. A.** Antibody labelling of A1ATD-iPSC patient 1 cells for OCT4 and NANOG, with DAPI counterstain, after plating on the FN concentrations shown. Cells were fixed after 1, 2 or 3 days. Scale bar: 150 µm. **B.** Q-PCR of OCT4 expression in FFDC_11 cells 24h after plating on the FN concentrations shown. Means ± SD of triplicate wells. **C, D.** A1ATD-iPSC patient 1 cells (C) or CTR M205 cells (D) were plated on vitronectin (C) or the FN concentrations shown (D) for 24h and then harvested and replated on feeders (D) or under feeder-free conditions (C). Two days later the cultures were fixed and labelled with antibodies to NANOG and OCT4 with DAPI counterstain. Representative colonies are shown. The same colonies are shown in each panel of (C). Scale bar: 150 µm.

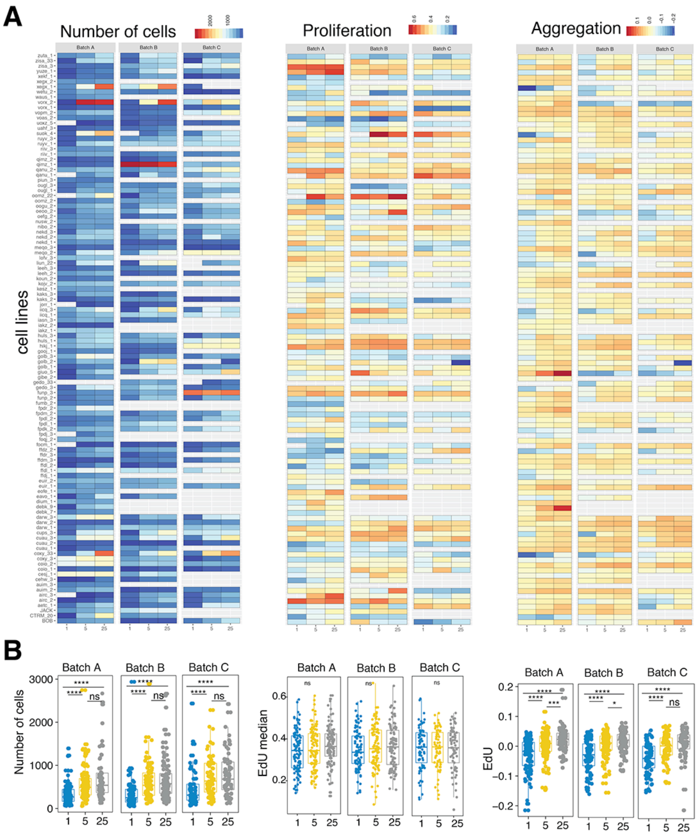


**Figure S2. Related to Figure 1. A.** Heatmaps of mean values for three phenotypic measurements: number of cells, proliferation (EdU incorporation) and cell aggregation (clump size) for each cell line on three fibronectin concentrations in three independent experiments (batches). Grey boxes correspond to experiments not performed. **B.** Boxplots of mean values for the same three phenotypic measurements on three fibronectin concentrations in three biological replicates (batches). Each dot is one cell line. Asterisks (**** P ≤ 0.0001; *** P≤ 0.001; ** P ≤ 0.01; * P ≤ 0.05; ns not significant) represent significance values from pairwise t-tests performed between each condition.

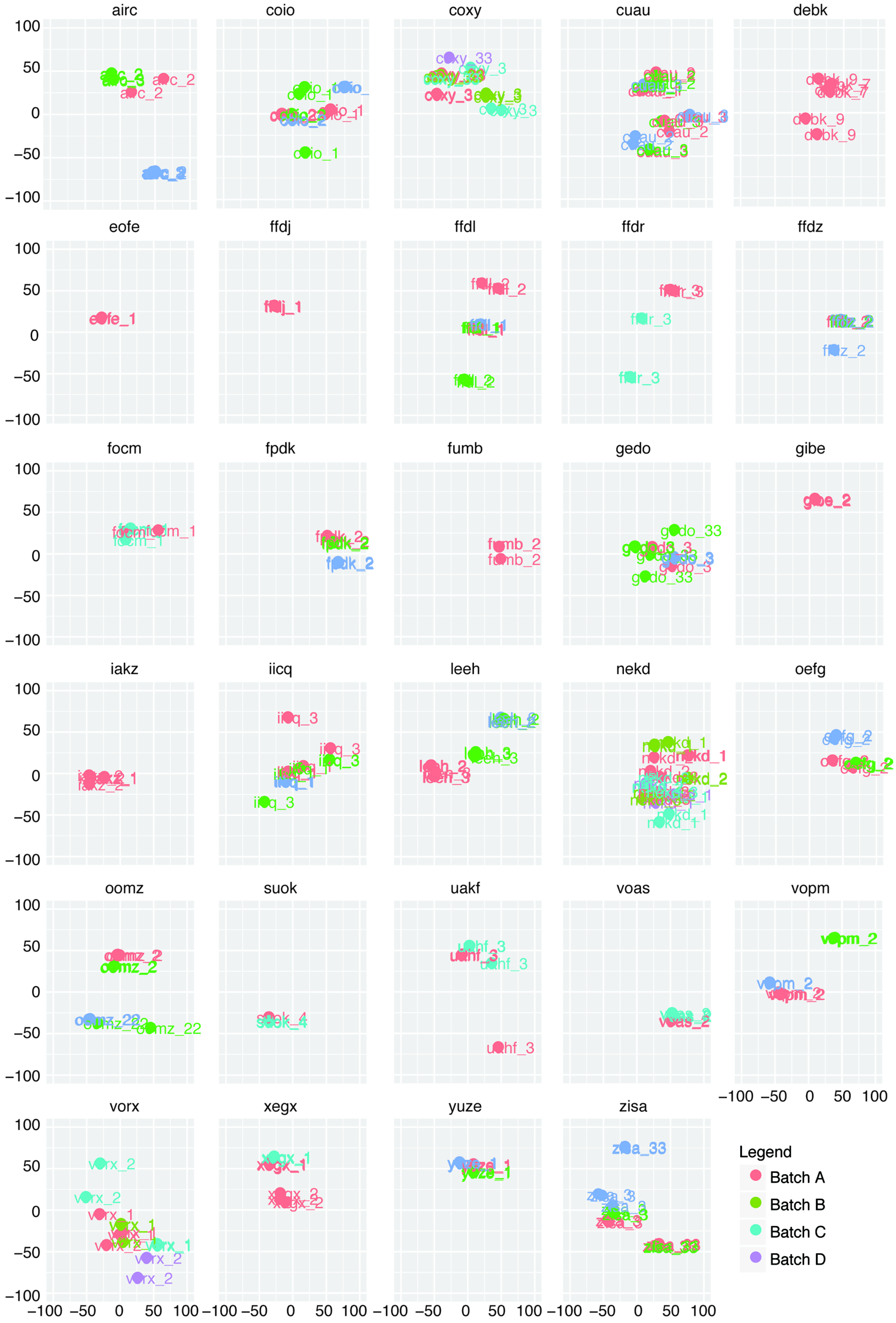


**Figure S3. Related to Figure 1.** t-SNE plots for each outlier cell line representing the reproducibility (cell area phenotype) of 3 technical replicates per batch, and variation between batches. Each dot within each plot is a technical replicate. Colors represent individual batches. Different lines from the same donor are also indicated.

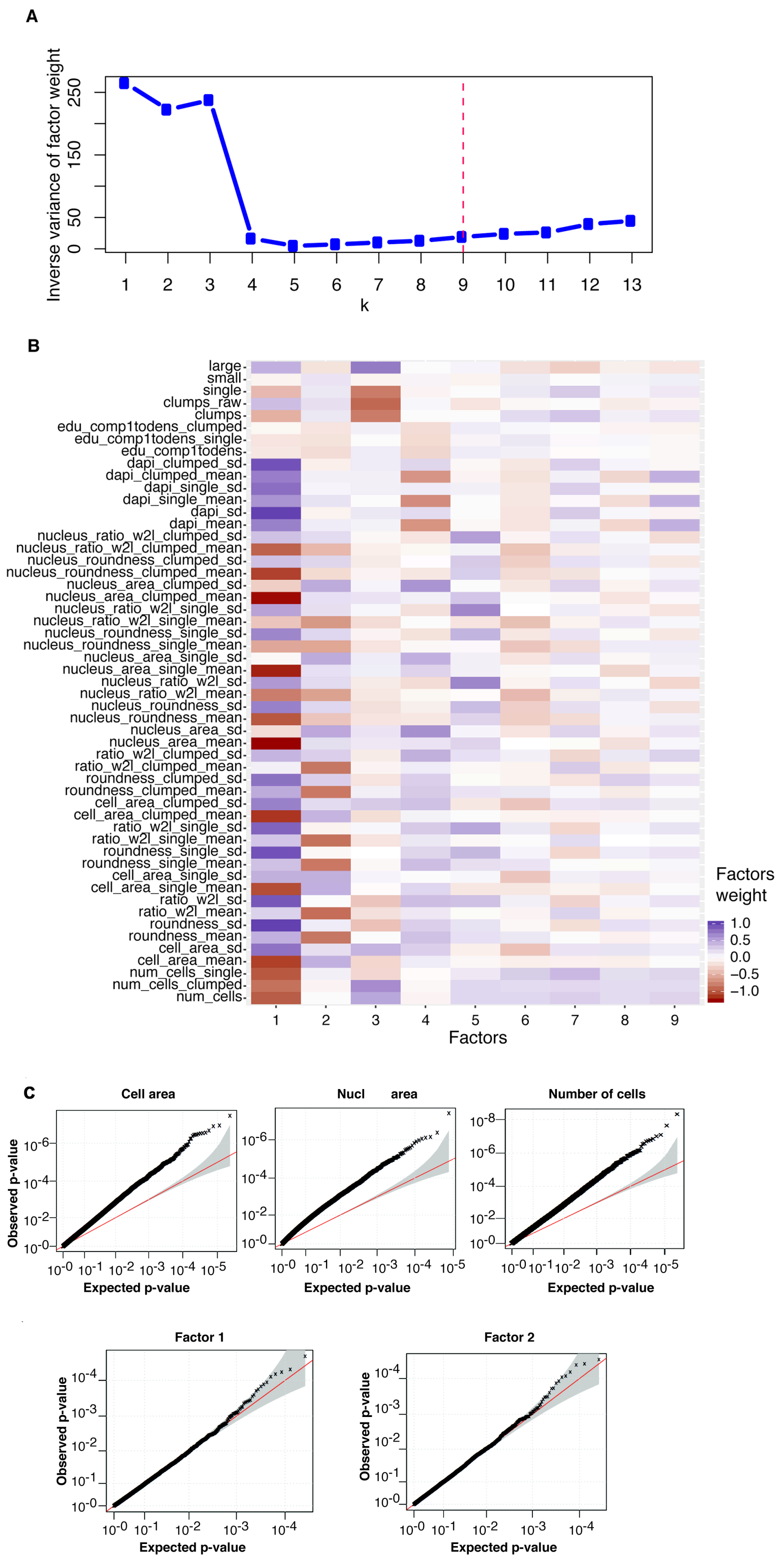


**Figure S4. Related to Figure 3. A.** Plot of inverse variance of PEER factor weights (y axis) against k number (x axis) (usually observed as an "elbow"). **B.** Heatmap showing weight of each raw phenotype onto each of the 9 PEER factors. **C.** Q-Q plots in which the -log10 of p-values observed for the correlation analyses using raw data (upper panels) or the PEER factors (lower panels) are plotted against the theoretical -log10 p-values expected under the null hypothesis (red line). Each dot represents one gene.

**Table S2**

| | | | |
|---|---|---|---|
| Cell area | Clumped cells | mean | 1 |
| | | standard deviation | 2 |
| | Single cells | mean | 3 |
| | | standard deviation | 4 |
| | All cells | mean | 5 |
| | | standard deviation | 6 |
| Cell ratio w/l | Clumped cells | mean | 7 |
| | | standard deviation | 8 |
| | Single cells | mean | 9 |
| | | standard deviation | 10 |
| | All cells | mean | 11 |
| | | standard deviation | 12 |
| Cell roundness | Clumped cells | mean | 13 |
| | | standard deviation | 14 |
| | Single cells | mean | 15 |
| | | standard deviation | 16 |
| | All cells | mean | 17 |
| | | standard deviation | 18 |
| Nucleus area | Clumped cells | mean | 19 |
| | | standard deviation | 20 |
| | Single cells | mean | 21 |
| | | standard deviation | 22 |
| | All cells | mean | 23 |
| | | standard deviation | 24 |
| Nucleus ratio w/l | Clumped cells | mean | 25 |
| | | standard deviation | 26 |
| | Single cells | mean | 27 |
| | | standard deviation | 28 |
| | All cells | mean | 29 |
| | | standard deviation | 30 |
| Nucleus roundness | Clumped cells | mean | 31 |
| | | standard deviation | 32 |
| | Single cells | mean | 33 |
| | | standard deviation | 34 |
| | All cells | mean | 35 |
| | | standard deviation | 36 |
| DAPI | Clumped cells | mean | 37 |
| | | standard deviation | 38 |
| | Single cells | mean | 39 |
| | | standard deviation | 40 |
| | All cells | mean | 41 |
| | | standard deviation | 42 |
| EdU | Clumped cells | mean | 43 |
| | | standard deviation | 44 |
| | Single cells | mean | 45 |
| | | standard deviation | 46 |
| | All cells | mean | 47 |
| | | standard deviation | 48 |
| Number of cells | Clumped cells | | 49 |
| | Single cells | | 50 |
| | All cells | | 51 |
| Clumps | | | 52 |

**Table S2. Related to Figure 1.** List of all 52 cell features analysed.
